# Effects of Personalized Nursing Combined With Mindfulness-Based Stress Reduction on Anxiety, Depression, and Quality of Life in Patients With Pneumonia-Induced Sepsis

**DOI:** 10.62641/aep.v54i3.2212

**Published:** 2026-06-15

**Authors:** Xinrong Zheng, Qijun Zhang, Hesheng Zhuang

**Affiliations:** ^1^Department of General Practice, The People's Hospital of Pingyang, 325400 Wenzhou, Zhejiang, China; ^2^Department of Emergency Medicine, The People's Hospital of Pingyang, 325400 Wenzhou, Zhejiang, China; ^3^Department of Nursing, The People's Hospital of Cangnan, 325800 Wenzhou, Zhejiang, China

**Keywords:** personalized nursing, mindfulness-based stress reduction (MBSR), pneumonia-induced sepsis, anxiety, depression

## Abstract

**Background:**

This study aimed to investigate the effects of personalized nursing combined with Mindfulness-Based Stress Reduction (MBSR) on anxiety, depression, self-efficacy, quality of life and self-care ability in patients with pneumonia-induced sepsis.

**Methods:**

This retrospective study enrolled 127 patients with pneumonia-induced sepsis admitted between July 2023 to July 2024. Among them, 59 patients in the routine nursing group received routine nursing care, while 68 patients in the combined nursing group received personalized nursing combined with MBSR. Routine nursing included vital signs monitoring, oxygen therapy, medication management, basic daily care, nutritional support, health education and complication prevention. The combined nursing group additionally received personalized nursing (e.g., psychological assessment and support, targeted health education, and dynamic adjustment of care plans) and MBSR training (e.g., mindful breathing, body scanning, mindfulness meditation and relaxation training), delivered daily or several times per week until discharge. Outcome measures included the Hamilton Anxiety Scale (HAMA), Hamilton Depression Scale (HAMD), General Self-Efficacy Scale (GSES), World Health Organization Quality of Life-100 (WHOQOL-100), and Exercise of Self-Care Agency (ESCA), assessed before and after nursing measures. Categorical variables were compared using the chi-square test, and continuous variables (mean ± standard deviation) were analysed using independent-samples *t* tests.

**Results:**

No statistically significant baseline differences in demographic or clinical characteristics were observed between the two groups (*p* > 0.05). After nursing measures, both groups showed improvements in HAMA, HAMD, GSES, WHOQOL-100, and ESCA scores compared with baseline. The combined nursing group demonstrated significantly greater reduction in HAMA and HAMD scores (*p* < 0.05) and significantly greater increases in GSES, WHOQOL-100, and ESCA scores (*p* < 0.05) than the routine nursing group.

**Conclusions:**

An integrated approach combining personalized nursing and MBSR was associated with lower levels of negative emotional states, higher self-efficacy in disease management, and better quality of life and self-care ability in patients with pneumonia-induced sepsis. These findings suggest that this nursing model may represent a feasible and potentially beneficial strategy for improving psychological and functional outcomes in clinical practice.

## Introduction

Pneumonia-induced sepsis is a common and critical condition among severely ill patients, with high morbidity and mortality rate, particularly pronounced in elderly individuals and those with underlying comorbidities [1, 2, 3]. Sepsis initially triggers severe systemic inflammatory responses and immune dysfunction, which can subsequently progress to multiple organ dysfunction, thereby prolonging Intensive Care Unit (ICU) stay and mechanical ventilation duration, increasing medical burden and complication risks [4, 5].

Beyond the substantial physiological burden, patients often experience considerable psychological stress during the disease course. Studies have shown that patients with pneumonia-induced sepsis are susceptible to adverse emotional states, including anxiety and depressive symptoms during hospitalization. These symptoms not only affect sleep quality and life satisfaction but may also reduce treatment adherence, exacerbate disease progression, and delay recovery [6, 7]. Therefore, effectively alleviating patients’ psychological stress and improving their self-efficacy and quality of life have become an important goal in clinical nursing practice.

Traditional nursing primarily focuses on monitoring vital signs, airway management, medication therapy, basic daily care, nutritional support and complication prevention. Although traditional nursing address patients’ physiological needs, it remains insufficient in terms of psychological measures and comprehensive functional improvement [8]. With the development of psychological nursing concepts, researchers have gradually recognized routine nursing alone is inadequate to meet the mental health needs of patients with sepsis [9, 10].

Mindfulness-Based Stress Reduction (MBSR) is a structured psychological measure grounded in mindfulness that guides individuals to focus on the present moment and observe their emotions and bodily sensations with a nonjudgmental attitude [11, 12]. MBSR helps alleviate anxiety and depression and improves emotional regulation and psychological resilience. In patients with various chronic diseases and critical illnesses, MBSR has been shown to enhance self-efficacy, strengthen coping abilities under stress, and improve quality of life [13, 14]. Integrating MBSR into nursing care can provide systematic psychological support and compensate for the limitations of routine nursing in psychological care.

Personalized nursing emphasises formulating individualized care plans based on patients’ disease characteristics, psychological status, and lifestyle habits, and providing targeted health education, emotional support, and lifestyle guidance [15]. The integration of personalized nursing with MBSR addresses both physical rehabilitation and psychological well-being, thereby achieving a holistic and systematic nursing approach. However, research on the application of personalized nursing combined with MBSR in patients with pneumonia-induced sepsis remains limited, particularly regarding comprehensive evaluation of anxiety, depression, self-efficacy, self-care ability, and quality of life.

Therefore, this study aimed to investigate the effects of personalized nursing combined with MBSR measures on anxiety, depression, self-efficacy, quality of life, and self-care ability in patients with pneumonia-induced sepsis, in order to provide feasible measures strategies and evidence-based support for clinical nursing practice, while enriching the application of psychological nursing in critically ill patients’ management.

## Materials and Methods

### General Information

This study was a retrospective comparative study conducted at a single institution. A total of 127 patients diagnosed with sepsis secondary to pneumonia and hospitalized at The People’s Hospital of Pingyang between July 2023 and July 2024 were included. Based on the nursing approaches documented in clinical records, participants were allocated to either a routine nursing group (n = 59) or a combined nursing group (n = 68). Baseline demographic and clinical variables were retrospectively extracted from the hospital’s electronic health record database. Patients were grouped according to the actual nursing care received in routine clinical practice. Ethical approval for this study was granted by the Ethics Committee of The People’s Hospital of Pingyang (IRB-2024-017). Owing to the retrospective nature of the study, all data were retrieved from existing electronic medical records. To ensure confidentiality, patient identifiers were removed during data processing, and privacy was fully safeguarded. The study protocol complied with the principles of the Declaration of Helsinki and applicable ethical standards. Written informed consent had been obtained from all participants.

#### Inclusion Criteria

(1) Patients who met the diagnostic criteria for pneumonia-induced sepsis (pneumonia confirmed as the primary infection source, and diagnosis meeting Sepsis-3 criteria with infection-related organ dysfunction [16]);

(2) those with complete medical records and nursing documentation clearly indicating the nursing model;

(3) patients who were conscious, had basic communication ability, and could cooperate with the assessment of anxiety, depression, and quality-of-life scales;

(4) age ≥ 18 years.

#### Exclusion Criteria

(1) Patients with severe mental disorders or a prior history of psychiatric illness;

(2) those with malignant tumours, end-stage organ failure, or other severe systemic diseases that could severely affect prognosis and quality of life;

(3) patients with severe cognitive impairment or consciousness disturbance who were unable to complete the questionnaires;

(4) pregnant or lactating women;

(5) patients who had received systematic MBSR training or other psychological measures before admission.

### Selection Process

A total of 185 patients with pneumonia-induced sepsis were initially assessed for eligibility between July 2023 and July 2024. Among them, 32 patients were excluded due to severe comorbid conditions or cognitive impairment, and 26 patients were excluded because of incomplete clinical data. Ultimately, 127 patients were included in the final analysis.

### Methods

#### Routine Nursing Group (Routine Nursing)

Patients in the routine nursing group received routine nursing care, which specifically included:

(1) Condition observation and monitoring: Patients’ core vital parameters—body temperature, cardiac rhythm, respiratory pattern, blood pressure and oxygen saturation—were subject to regular surveillance. Any abnormalities were promptly reported to the physician, and appropriate assistance was provided.

(2) Oxygen therapy and airway care: Oxygen therapy was administered according to the patient’s condition. Nurses assisted with sputum expectoration, maintained airway patency, and guided patients to perform effective coughing and deep-breathing exercises.

(3) Medication nursing: Medical orders were strictly implemented. Anti-infective, antipyretic, vasopressor, and other symptomatic and supportive medications were administered accurately and on time, with close observation of therapeutic effects and potential adverse reactions.

(4) Basic daily care: Patients were assisted with activities of daily living. Skin and oral hygiene were maintained, regular repositioning was performed to prevent pressure injuries, and a clean and comfortable ward environment was ensured.

(5) Nutritional support nursing: Appropriate dietary guidance was provided based on the patient’s condition. A high-protein, high-calorie, and easily digestible diet was encouraged. Assistance with feeding or enteral nutritional support was provided when necessary.

(6) Health education: Basic education regarding the disease and treatment was provided to patients and their families to improve adherence treatment and nursing care.

(7) Complication prevention: Routine nursing measures were implemented to prevent common complications such as pressure injuries, deep vein thrombosis and nosocomial infections.

#### Combined Nursing Group (Personalized Nursing Combined With Mindfulness-Based Stress Reduction)

On the basis of routine nursing, patients in the combined nursing group received personalized nursing combined with MBSR. The specific measures were as follows:

(1) Comprehensive individualized assessment: After admission, the responsible nurse systematically assessed the severity of the patient’s condition, psychological status, sleep quality, anxiety and depression levels, coping styles, social support, and formulated an individualized nursing care plan.

(2) Personalized nursing care: Individualized nursing goals were established according to the patient’s clinical condition and psychological characteristics. Through patient listening and empathetic communication, patients were encouraged to express their inner feelings, and timely psychological comfort and support were provided. Nurse–patient communication was strengthened to enhance patients’ sense of security and treatment adherence.

(3) Health education and cognitive measures: Information about pneumonia-induced sepsis, treatment procedures, and prognosis was explained to patients in a simple and understandable manner. Misconceptions about the disease were corrected to reduce fear and helplessness, and patients’ confidence in overcoming the illness was enhanced.

(4) MBSR training: Under the guidance of trained nursing staff, patients were instructed to perform mindfulness training, mainly including:

Mindful breathing training: guiding patients to focus their attention on the rhythm of breathing and perform slow and deep breathing.

Body scan: guiding patients to sequentially focus on sensations in different body parts to relieve tension and discomfort.

Mindfulness meditation: helping patients focus on the present moment and observe their emotions and thoughts with a nonjudgmental attitude.

Mindful relaxation training: combining breathing with muscle relaxation to alleviate psychological stress.

Patients received MBSR training once daily for 15–30 minutes per session until discharge.

(5) Emotional management and psychological support: Patients’ anxiety and depression levels were regularly assessed. Targeted psychological counselling was provided to those with prominent negative emotions. Family members were encouraged to participate in the nursing process to enhance patients’ social support.

(6) Sleep and lifestyle guidance: Patients were guided to establish regular daily routines. The ward environment was optimized by reducing noise and disturbances. Patients were instructed to apply mindfulness techniques to improve sleep quality.

(7) Dynamic evaluation and adjustment of measures: Changes in patients’ psychological status and quality of life were continuously monitored, and nursing plans and mindfulness measures were adjusted in a timely manner according to the assessment results.

The duration of nursing care in both groups corresponded to the length of hospitalization.

### Baseline Disease Severity Indicators

Baseline demographic and clinical information, including age, sex, medical history, disease severity indicators, duration of mechanical ventilatory, and duration of ICU stay, were retrospectively extracted from the hospital electronic medical record system.

Disease severity was evaluated using the Acute Physiology and Chronic Health Evaluation II (APACHE II) score [17] and the Sequential Organ Failure Assessment (SOFA) score [18]. The APACHE II score consists of 12 acute physiological variables, age, and chronic health status, with a total score ranging from 0 to 71; higher scores indicate more severe illness and a higher mortality risk. The APACHE II scoring system is widely used in intensive care settings to assess disease severity and predict prognosis, with good reliability and validity (Area Under the Curve (AUC) generally ranging from 0.75 to 0.90).

The SOFA score quantifies organ impairment by evaluating six domains: pulmonary function, coagulation status, hepatic function, cardiovascular function, neurological status, and renal function. Each component is graded 0 to 4, yielding a cumulative score of 0 to 24, higher scores reflect greater organ failure severity and worsen prognosis. The SOFA score is extensively utilized in sepsis for clinical diagnosis, severity classification, and prognosis estimation.

### Outcome Measures

The outcome measures included anxiety and depression, self-efficacy, quality of life, and self-care ability, as detailed below:

(1) Anxiety and depression were assessed using the Hamilton Anxiety Scale (HAMA) [19] and the Hamilton Depression Scale (HAMD) [20]. The HAMA consists of 14 items evaluating somatic and psychological anxiety symptoms, each rated 0–4, total score 0–56; higher scores indicate greater anxiety severity. The HAMD contains 17 items assessing depressive symptoms, scored on 2-point or 4-point scale, total score 0–52; higher scores indicate greater depression severity. Both scales demonstrate good internal consistency (Cronbach’s α = 0.80–0.90) and test–retest reliability (r = 0.82–0.91), and they are widely used in clinical and nursing research.

(2) Self-efficacy was evaluated using the General Self-Efficacy Scale (GSES) [21, 22]. The scale includes 10 items across three domains: positive orientation, autonomous decision-making, and self-regulation. Responses use a four-point Likert scale, total scores range from 10 to 40, higher scores reflect greater perceived self-efficacy. The GSES has satisfactory psychometric properties (Cronbach’s α = 0.85, r = 0.88).

(3) Quality of life was assessed using the World Health Organization Quality of Life-100 (WHOQOL-100) [23], which covers six dimensions: physical function, psychological capacity, social relationships, spirituality/beliefs, environmental status, and level of independence, comprising 100 items. Each item is rated on a 1–5 Likert scale; higher scores indicate better quality of life. The WHOQOL-100 has high internal consistency (Cronbach’s α = 0.88–0.92) and good structural and criterion-related validity.

(4) Self-care ability was evaluated using the Exercise of Self-Care Agency (ESCA) scale [24], a 43-item instrument comprising four domains: self-perception, personal responsibility, practical self-care skills, and health-related knowledge. Responses use a five-point Likert scale, total score ranges from 0 to 172, higher scores indicate greater self-care capacity. The ESCA scale demonstrates strong internal reliability (Cronbach’s α = 0.87) and sound validity.

All scale assessments were conducted before the nursing measures and after its completion.

### Statistical Analysis

Categorical variables were expressed as frequencies and percentages and compared using the chi-square test. The Shapiro–Wilk test indicated that all continuous variables followed a normal distribution; therefore, data were presented as mean ± standard deviation and analysed using independent-samples *t* tests for between-group comparisons. A two-sided *p* value < 0.05 was considered statistically significant.

## Results

### Comparison of Baseline Demographic and Clinical Variables Between the Two Groups

Comparative analysis revealed no significant differences between the two groups in demographic factors, comorbidities, or clinical severity indicators, including age, sex distribution, histories of hypertension, diabetes, coronary heart disease, and chronic obstructive pulmonary disease, as well as APACHE II and SOFA scores, duration of mechanical ventilatory, and duration of ICU hospitalization (all *p* values > 0.05, Table 1).

**Table 1.** **Comparison of baseline demographic and clinical variables between the two groups**.

Baseline demographic and clinical variables		Routine nursing group (n = 59)	Combined nursing group (n = 68)	*t*/χ^2^	*p*
Age (years old)		62.21 ± 10.44	61.57 ± 9.85	0.353	0.725
Gender (n/%)				0.412	0.521
	Male	27 (45.76)	35 (51.47)		
	Female	32 (54.24)	33 (48.53)		
History of hypertension (n/%)				0.005	0.944
	Yes	30 (50.85)	35 (51.47)		
	No	29 (49.15)	33 (48.53)		
History of diabetes (n/%)				0.108	0.742
	Yes	33 (55.93)	40 (58.82)		
	No	26 (44.07)	28 (41.18)		
History of coronary heart disease (n/%)				0.380	0.538
	Yes	28 (47.46)	36 (52.94)		
	No	31 (52.54)	32 (47.06)		
History of COPD (n/%)				0.027	0.869
	Yes	39 (66.10)	44 (64.71)		
	No	20 (33.90)	24 (35.29)		
APACHE-II scores		9.36 ± 1.87	9.35 ± 2.23	0.027	0.979
SOFA scores		9.24 ± 3.24	8.91 ± 2.93	0.602	0.548
Duration of mechanical ventilation (Days)		9.24 ± 3.24	9.29 ± 3.21	0.082	0.935
Duration of ICU stay (Days)		16.17 ± 4.67	16.37 ± 4.54	0.243	0.808

Note: COPD, chronic obstructive pulmonary disease; APACHE-II, Acute Physiology and Chronic Health Evaluation II; SOFA, Sequential Organ Failure Assessment; ICU, Intensive Care Unit.

### Comparison of Anxiety and Depression Scores Between the Two Groups

At baseline, no significant differences were observed in HAMA or HAMD scores between the two groups (*p* > 0.05). After the nursing measures, both groups showed reduction in anxiety and depression scores compared with pre-nursing levels. Notably, the combined nursing group achieved significantly lower HAMA and HAMD scores than the routine nursing group, with statistically meaningful differences (*p* < 0.05, Table 2).

**Table 2.** **Comparison of anxiety and depression scores between the two groups**.

Psychological scale	Routine nursing group (n = 59)	Combined nursing group (n = 68)	*t*	*p*
HAMA score before nursing	26.17 ± 4.67	26.54 ± 4.58	0.446	0.657
HAMA score after nursing	20.86 ± 4.70	18.14 ± 4.27	3.411	<0.001
HAMD score before nursing	21.58 ± 5.83	21.27 ± 4.57	0.336	0.737
HAMD score after nursing	19.70 ± 5.82	16.34 ± 5.27	3.418	<0.001

HAMA, Hamilton Anxiety Scale; HAMD, Hamilton Depression Scale.

### Comparison of Self-Efficacy Between the Two Groups

At baseline, no statistically significant differences were identified between the two groups in GSES domain scores, including positive outlook, autonomous decision-making and stress management (*p* > 0.05). After the nursing measures, improvements were observed across all three domains in both groups compared with baseline. Post-nursing comparisons demonstrated that the combined nursing group achieved significantly higher scores in all three domains (e.g., positive attitude, self-decision capacity and stress reduction ability) than the routine nursing group (*p* < 0.05, Table 3).

**Table 3.** **Comparison of GSES scores between the two groups**.

GSES	Routine nursing group (n = 59)	Combined nursing group (n = 68)	*t*	*p*
Positive attitude before nursing	23.99 ± 4.94	24.79 ± 4.93	0.909	0.365
Positive attitude after nursing	32.31 ± 5.49	36.61 ± 3.52	5.333	<0.001
Self-decision-making before nursing	23.53 ± 3.60	23.35 ± 3.67	0.277	0.783
Self-decision-making after nursing	29.54 ± 3.58	35.08 ± 4.90	7.163	<0.001
Self-stress reduction before nursing	27.99 ± 4.99	28.98 ± 4.94	1.120	0.265
Self-stress reduction after nursing	32.17 ± 5.65	35.64 ± 4.50	3.850	<0.001

GSES, General Self-Efficacy Scale.

### Comparison of Quality of Life Between the Two Groups

At baseline, there were no statistically significant differences between the two groups in WHOQOL-100 domain scores, including physical functioning, psychological capacity, social functioning, spirituality/religion, environmental status, and level of independence. (*p* > 0.05). After nursing, both groups showed higher scores than before. Moreover, the combined nursing group had significantly higher scores in all six domains than the routine nursing group (*p* < 0.05, Table 4).

**Table 4.** **Comparison of WHOQOL-100 scores between the two groups of patients**.

WHOQOL-100	Routine nursing group (n = 59)	Combined nursing group (n = 68)	*t*	*p*
Physical functioning before nursing	80.01 ± 4.94	80.56 ± 4.58	0.640	0.523
Physical functioning after nursing	83.12 ± 4.89	87.62 ± 4.49	5.399	<0.001
Psychological functioning before nursing	77.89 ± 4.90	77.79 ± 5.03	0.105	0.917
Psychological functioning after nursing	82.47 ± 6.95	86.86 ± 5.08	4.100	<0.001
Social functioning before nursing	74.88 ± 4.90	75.19 ± 5.49	0.331	0.741
Social functioning after nursing	81.53 ± 5.27	85.36 ± 4.78	4.295	<0.001
Spirituality/Beliefs before nursing	81.60 ± 5.28	82.19 ± 5.57	0.601	0.549
Spirituality/Beliefs after nursing	86.21 ± 5.23	88.38 ± 5.50	2.262	0.025
Environmental status before nursing	80.52 ± 6.87	80.98 ± 6.77	0.381	0.704
Environmental status after nursing	84.54 ± 6.23	87.77 ± 6.14	2.941	0.004
Level of Independence before nursing	53.97 ± 5.19	55.54 ± 5.00	1.735	0.085
Level of Independence after nursing	65.06 ± 4.52	72.63 ± 4.73	9.179	<0.001

Note: WHOQOL-100, World Health Organization Quality of Life-100.

### Comparison of Self-Care Ability Between the Two Groups

At baseline, no significant differences were detected between the two groups in ESCA domain scores, including self-perception, responsibility for self-care, practical self-care abilities, and health-related knowledge (*p* > 0.05). After the nursing measures, both groups exhibited improvements across all ESCA dimensions compared with baseline. Importantly, the combined nursing group achieved significantly higher scores in all four domains than the routine nursing group (*p* < 0.05, Table 5).

**Table 5.** **ESCA scores of the two groups of patients**.

ESCA	Routine nursing group (n = 59)	Combined nursing group (n = 68)	*t*	*p*
Self-concept before nursing	9.30 ± 2.34	8.98 ± 2.12	0.82	0.414
Self-concept after nursing	23.70 ± 4.93	27.65 ± 4.78	4.573	<0.001
Sense of self-responsibility before nursing	9.81 ± 1.61	10.11 ± 1.93	0.95	0.344
Sense of self-responsibility after nursing	17.01 ± 2.31	20.62 ± 2.34	8.724	<0.001
Self-care skills before nursing	22.70 ± 4.97	23.20 ± 5.24	0.545	0.587
Self-care skills after nursing	34.51 ± 5.25	43.43 ± 3.74	11.129	<0.001
Health knowledge before nursing	23.79 ± 5.70	23.96 ± 5.12	0.185	0.854
Health knowledge after nursing	53.79 ± 7.52	59.34 ± 6.42	4.489	<0.001

ESCA, Exercise of Self-Care Agency.

## Discussion

This study found that implementing personalized nursing combined with MBSR was associated with lower levels of anxiety and depression in patients with pneumonia-induced sepsis, as well as with higher self-efficacy, self-care ability, and quality of life. Compared with routine nursing alone, the combined nursing group showed more favourable scores across all assessment scales after the measures. These findings indicate that integrating of psychological nursing measures and with individualized care is associated with improved patient-reported outcomes and may represent a potentially beneficial approach in critically ill patients’ management.

Regarding anxiety and depression, the findings are consistent with previous reports. Existing literature has shown that patients with sepsis often experience considerable psychological stress due to disease severity, prognosis uncertainty, and prolonged ICU treatment, resulting in a high incidence of anxiety and depression [25, 26]. In this study, MBSR effectively alleviated anxiety and depression by guiding patients to focus on the present moment, become aware of their own emotions, and reduce negative expectations about their illness and the future. This aligns with the findings of Chen *et al*. [27], who applied mindfulness-based measures in ICU patients and demonstrated remarkably improvements in anxiety and depressive symptoms. Additionally, integrating individualized nursing strategies—including psychological comfort, emotional counselling, and health education—further enhanced patients’ sense of security and confidence, thereby strengthening the overall effect of the psychological measures.

In terms of self-efficacy and self-care ability, this study found that GSES and ESCA scores in the combined nursing group were higher after the nursing measures. This pattern is consistent with both domestic and international studies reporting that psychological treatments are associated with improved self-management abilities in critically ill patients. An eight-week mindfulness training program integrated into primary care was more effective than a low-dose comparator in facilitating chronic disease self-management behaviours among patients with anxiety, depression, and stress-related disorders, suggesting enhanced self-management capacity with mindfulness training [28]. In the present study, personalized nursing was applied to assess patients’ psychological status and needs, and was combined with MBSR training. This approach was associated with greater patient participation in their own health management during the nursing process and with higher levels of self-efficacy and self-care ability.

Regarding quality of life, this study showed that patients in the combined nursing group had higher scores across all WHOQOL-100 dimensions than those in the routine nursing group. A previous study has shown that comprehensive psychological nursing measures in critically ill patients can improve their physical, psychological, social, and environmental adaptation abilities, thereby enhancing overall quality of life [29]. Notably, the present study combined mindfulness training with sleep and lifestyle guidance, which may have exerted a synergistic effect on improving sleep quality and emotional regulation, thereby enhancing overall quality of life.

The results of this study indicate that personalized nursing combined with MBSR is associated with multidimensional improvements in patient outcomes. lower levels of negative emotions, higher self-efficacy and self-care ability in coping with disease, and better quality of life. Compared with psychological measures alone or routine nursing, this combined approach was associated with more favourable overall outcome profiles. Unlike the previous study that focused primarily on anxiety or depression, this study systematically assessed psychological, behavioural, and quality-of-life indicators, thereby providing a more comprehensive description of the observed nursing-related outcomes.

This study has several limitations. Firstly, the design of the current study was retrospective and non-randomized, which limited the ability to establish causal relationships and may introduce selection bias and unmeasured confounding, despite comparable baseline characteristics. Secondly, the statistical analysis was limited to between-group comparisons at each time point; within-group paired analyses, change score comparisons, and adjusted analyses such as analysis of covariance or multivariable regression were not performed, which may reduce the robustness and internal validity of the findings. Thirdly, the single-centre design with a relatively small sample size may limit the generalizability of the results. Fourthly, outcome measures—including psychological status, self-efficacy, quality of life, and self-care ability—were based on subjective questionnaires, which are susceptible to reporting bias. Finally, the lack of long-term follow-up precludes assessment of the durability of the observed effects. Therefore, the findings should be interpreted as associative rather than causal, and future large-scale, multicentre randomized controlled trials with standardized analytical methods and extended follow-up are needed for validation.

## Conclusions

Personalized nursing combined with MBSR measures was significantly associated with reduced anxiety and depressive symptoms, as well as improved self-efficacy, self-care ability, and quality of life in patients with pneumonia-induced sepsis. These findings suggest that this approach may represent a feasible and comprehensive strategy for the nursing care of critically ill patients.

## Availability of Data and Materials

The analyzed data sets generated during the study are available from the corresponding author upon reasonable request.

## References

[1] Wang C, Zhao D, Zheng L, Bao X, Yang Q, Jiang S (2022). Safety and efficacy of human umbilical cord mesenchymal stem cells for the treatment of sepsis induced by pneumonia: study protocol for a single-centre, randomised single-blind parallel group trial. *BMJ open*.

[2] Wu J, Li X, Chen Z, Lin Y, Long Q, Jiang M (2025). Sphingolipid metabolism-related genes as diagnostic markers in pneumonia-induced sepsis: the AUG model. *Scientific reports*.

[3] Wei Z, YuSuPu M, Wang X, Meng Y, Zhang C, Xue X (2025). The accuracy of heparin-binding protein and interleukin-6 in predicting prognosis of severe pneumonia with sepsis patients. *Frontiers in cellular and infection microbiology*.

[4] Wang T, Zhang X, Liu Z, Yao T, Zheng D, Gan J (2021). Single-cell RNA sequencing reveals the sustained immune cell dysfunction in the pathogenesis of sepsis secondary to bacterial pneumonia. *Genomics*.

[5] Darkwah S, Kotey FCN, Ahenkorah J, Adutwum-Ofosu KK, Donkor ES (2024). Sepsis-Related Lung Injury and the Complication of Extrapulmonary Pneumococcal Pneumonia. *Diseases (Basel, Switzerland)*.

[6] Calsavara AJ, Costa PA, Nobre V, Teixeira AL (2021). Prevalence and risk factors for post-traumatic stress, anxiety, and depression in sepsis survivors after ICU discharge. *Revista brasileira de psiquiatria (São Paulo, Brazil : 1999)*.

[7] Wu CR, Zhu HL, Sun YT, Shen SH, Shi PL, Cui YH (2025). Clinical manifestations of anxiety and depression in sepsis-associated encephalopathy and multi-omics identification of cluster of differentiation 38 as an early biomarker. *World journal of psychiatry*.

[8] Dierkes AM, Aiken LH, Sloane DM, McHugh MD (2021). Association of hospital nursing and postsurgical sepsis. *PloS one*.

[9] Fan M, Zhu S, Liu M, Cao A, Jiang F, Wang R (2024). Nursing Based on Humanistic Care Concept for Continuous Blood Purification for Patients with Severe Sepsis in the Intensive Care Unit. *Alternative therapies in health and medicine*.

[10] Shen G, He Y, Ni J, Jiang L, Xia Z, Liu H (2021). Effects of comprehensive nursing on negative emotion and prognosis of patients with sepsis. *American journal of translational research*.

[11] (2023). Mindfulness-Based Stress Reduction for Nurses: An Integrative Review. *Journal of holistic nursing : official journal of the American Holistic Nurses’ Association*.

[12] Dong X, Liu Y, Fang K, Xue Z, Hao X, Wang Z (2024). The use of mindfulness-based stress reduction (MBSR) for breast cancer patients-meta-analysis. *BMC psychology*.

[13] Forte P, Abate V, Bolognini I, Mazzoni O, Quagliariello V, Maurea N (2023). Mindfulness-based stress reduction in cancer patients: impact on overall survival, quality of life and risk factor. *European review for medical and pharmacological sciences*.

[14] Tao S, Geng Y, Li M, Ye J, Liu Z (2022). Effectiveness of mindfulness-based stress reduction and mindfulness-based cognitive therapy on depression in poststroke patients-A systematic review and meta-analysis of randomized controlled trials. *Journal of psychosomatic research*.

[15] Lu L, Huang H (2025). Impact of Personalized Nursing Care on Bowel Preparation for Colonoscopy. *Journal of nursing care quality*.

[16] Singer M, Deutschman CS, Seymour CW, Shankar-Hari M, Annane D, Bauer M (2016). The Third International Consensus Definitions for Sepsis and Septic Shock (Sepsis-3). *JAMA*.

[17] Knaus WA, Draper EA, Wagner DP, Zimmerman JE (1985). APACHE II: a severity of disease classification system. *Critical care medicine*.

[18] Vincent JL, Moreno R, Takala J, Willatts S, De Mendonça A, Bruining H (1996). The SOFA (Sepsis-related Organ Failure Assessment) score to describe organ dysfunction/failure. On behalf of the Working Group on Sepsis-Related Problems of the European Society of Intensive Care Medicine. *Intensive care medicine*.

[19] HAMILTON M (1959). The assessment of anxiety states by rating. *The British journal of medical psychology*.

[20] HAMILTON M (1960). A rating scale for depression. *Journal of neurology, neurosurgery, and psychiatry*.

[21] Schwarzer R, Jerusalem M, Weinman J, Wright S, Johnston M (1995). Generalized self-efficacy scale. *Measures in health psychology: A user’s portfolio. Causal and control beliefs*.

[22] Schwarzer R, Mueller J, Greenglass E (1999). Assessment of perceived general self-efficacy on the internet: Data collection in cyberspace. *Anxiety, stress, and coping*.

[23] Kim JW, Lee YJ, Chung JW, Ha YS, Lee JN, Yoo ES (2018). Clinical characteristics of postoperative febrile urinary tract infections after ureteroscopic lithotripsy. *Investigative and clinical urology*.

[24] Wang T, Wang S, Wu N, Liu Y (2024). The mediating effect of self-efficacy on the relationship between self-care ability and disability level in older adult patients with chronic diseases. *Frontiers in public health*.

[25] Prescott HC, Angus DC (2018). Enhancing Recovery From Sepsis: A Review. *JAMA*.

[26] Wang Y, Zhu Y, Tian M, Wang Y, Pei X, Jiang J (2023). Recent advances in the study of sepsis-induced depression. *Journal of intensive medicine*.

[27] Chen Y, Wang R, Yu J, Zhu L, Lu Y, Deng X (2022). Effects of MBSR therapy on negative emotions, fatigue, and sleep quality in “post-ICU patients”: A randomized controlled clinical trial protocol. *Medicine*.

[28] Gawande R, To MN, Pine E, Griswold T, Creedon TB, Brunel A (2019). Mindfulness Training Enhances Self-Regulation and Facilitates Health Behavior Change for Primary Care Patients: a Randomized Controlled Trial. *Journal of general internal medicine*.

[29] Reibel DK, Greeson JM, Brainard GC, Rosenzweig S (2001). Mindfulness-based stress reduction and health-related quality of life in a heterogeneous patient population. *General hospital psychiatry*.

